# Usefulness of Intratracheal Instillation Studies for Estimating Nanoparticle-Induced Pulmonary Toxicity

**DOI:** 10.3390/ijms17020165

**Published:** 2016-01-27

**Authors:** Yasuo Morimoto, Hiroto Izumi, Yukiko Yoshiura, Kei Fujishima, Kazuhiro Yatera, Kazuhiro Yamamoto

**Affiliations:** 1 Department of Occupational Pneumology, Institute of Industrial Ecological Science, University of Occupational and Environmental Health, 1-1 Iseigaoka, Yahata-nishi-ku, Kitakyushu, Fukuoka 807-8555, Japan; h-izumi@med.uoeh-u.ac.jp (H.I.); y-yoshiura@med.uoeh-u.ac.jp (Y.Y.); fujishima_kei@med.uoeh-u.ac.jp (K.F.); 2 Department of Respiratory Medicine, University of Occupational and Environmental Health, 1-1 Iseigaoka, Yahata-nishi-ku, Kitakyushu, Fukuoka 807-8555, Japan; yatera@med.uoeh-u.ac.jp; 3 National Institute of Advanced Industrial Science and Technology (AIST), 1-1-1 Higashi, Tsukuba, Ibaraki 305-8565, Japan; k-yamamoto@aist.go.jp

**Keywords:** nanoparticle, intratracheal instillation, inhalation, harmful effect

## Abstract

Inhalation studies are the gold standard for the estimation of the harmful effects of respirable chemical substances, while there is limited evidence of the harmful effects of chemical substances by intratracheal instillation. We reviewed the effectiveness of intratracheal instillation studies for estimating the hazards of nanoparticles, mainly using papers in which both inhalation and intratracheal instillation studies were performed using the same nanoparticles. Compared to inhalation studies, there is a tendency in intratracheal instillation studies that pulmonary inflammation lasted longer in the lungs. A difference in pulmonary inflammation between high and low toxicity nanoparticles was observed in the intratracheal instillation studies, as in the inhalation studies. Among the endpoints of pulmonary toxicity, the kinetics of neutrophil counts, percentage of neutrophils, and chemokines for neutrophils and macrophages, heme oxygenase-1 (HO-1) in bronchoalveolar lavage fluid (BALF), reflected pulmonary inflammation, suggesting that these markers may be considered the predictive markers of pulmonary toxicity in both types of study. When comparing pulmonary inflammation between intratracheal instillation and inhalation studies under the same initial lung burden, there is a tendency that the inflammatory response following the intratracheal instillation of nanoparticles is greater than or equal to that following the inhalation of nanoparticles. If the difference in clearance in both studies is not large, the estimations of pulmonary toxicity are close. We suggest that intratracheal instillation studies can be useful for ranking the hazard of nanoparticles through pulmonary inflammation.

## 1. Introduction

In the rapid progress in nanotechnology, the development of new nanoparticle materials based on metals or carbon has advanced. However, neither the hazards nor a system to evaluate the hazards of these nanoparticles has been established, making it urgent to create a hazard evaluation system for the varieties of nanoparticles.

The gold standard study for estimating the harmful effects of respirable chemical substances is inhalaton studies, which give us extremely important information about the pulmonary toxicity of respirable chemical substances because the physiological exposure route is similar to that in humans [1]. But the high cost, facilities to house the large apparatus, and the mastery of skills of the handler are necessary, and it is virtually impossible to perform inhalation studies for all respirable chemical substances because there are so many of them.

The method in intratracheal instillation studies, on the other hand, is to expose the chemical substance directly through the trachea, and they are suitable for not only the elucidation of dose-response relations with a known quantity dosage but also for the elucidation of lung disorders induced by chemical substances. In addition, the expense is low, and large facilities are unnecessary. Therefore, the utilization of intratracheal instillation studies should be considered, although at present such studies have not provided enough evidence of the harmful effects of chemical substances, and their evaluation is restricted.

Here we review the similarities and differences in the data on the pulmonary toxicity of nanoparticles between inhalation and intratracheal instillation studies, and give suggestions on how to avoid the discrepancies in data between the two types of studies. It is thought that the reactivity of nanoparticles may be different between laboratories because there may be a difference in the physicochemical characteristics of the nanoparticles due to differences in the treatment process, even if the same raw materials are used [2,3,4]. For example, pulmonary inflammation varies according to difference in length even in the same multi-walled carbon nanotubes [4]. Therefore, we mainly reviewed references in which both inhalation and intratracheal instillation studies were performed using the same nanoparticles (Table 1), and reviewed the similarities and differences in the data on the pulmonary toxicity (Table 2). The papers in which animals were used have shown documentation of animal protection.

**Table 1:** Main references in which both inhalation and intratracheal instillation studies of nanoparticles were performed.

Nanoparticle	Animal	Experimental Design	Results	References
TiO_2_	F-344 rat	Inhalation Exposure: 13, 33 mg/m^3^ for 4 days Recovery: 4 h–7 days Intratracheal Exposure: 40–180 μg/rat Recovery: 4 h–7 days	Inflammation Inhalation: Transient increase Intratracheal: Transient increase Chemokine Inhalation: No response Intratracheal: Persistent increase Oxidative stress Inhalation: No response Intratracheal: Persistent increase	[5]
TiO_2_: Low toxicity	F-344 rat	Inhalation Exposure: 2 mg/m^3^ for 1 month Recovery: 3 days–3 months Intratracheal Exposure: 200, 1000 μg/rat Recovery: 3 days–6 months	Inflammation Inhalation: NiO, CeO_2_: Increase TiO_2_: No response Intratracheal: NiO CeO_2_: Persistent increase TiO_2_: Transient increase Chemokines Inhalation: NiO, CeO_2_: Increase TiO_2_: No response Intratracheal: NiO, CeO_2_: Persistent increase TiO_2_: Transient increase Oxidative stress Inhalation: NiO, CeO_2_: Increase TiO_2_: No response Intratracheal: NiO, CeO_2_: Persistent increase TiO_2_: Transient increase	[6]
NiO: High toxicity				
CeO_2_: High toxicity	F-344 rat	Inhalation Exposure: 2, 10 mg/m^3^ for 1 month Recovery: 3 days–3 months Intratracheal Exposure: 200, 1000 μg/rat Recovery: 3 days–6 months		[7]
MWCNT	SD rat	Inhalation Exposure: 30 mg/m^3^ for 6 h Recovery: 1–21 days Intratracheal Exposure: 10, 50, 200 μg/rat Recovery: 1–21 days	Inflammation Inhalation: No response Intratracheal: Transient increase	[8]
SWCNT	C57BL/6 mice	Inhalation Exposure 5 mg/m^3^ for 4 days Recovery: 1–28 days Aspiration Exposure: 5–20 μg/rat Recovery: 1–21 days	Inflammation Inhalation: Persistent increase Aspiration: Transient tendency Collagen Inhalation: Persistent increase Aspiration: Transient tendency	[9]

**Table 2:** Similarities and differences between intratracheal instillation and inhalation studies of data at endpoint.

Endpoint	Similarities between both Studies	Differences between both Studies
Total cell counts in BALF	Upregulation by nanoparticles Some of nanoparticles with high toxicity did not	Inflammatory quantity: IT study ≥ IH study Persistency of inflammation: IT study ≥ IH study
Number of Neutrophils in BALF	Upregulation by nanoparticles	
Percentage of neutrophils in BALF	Upregulation by nanoparticles	
Proinflammaotry cytokines in BALF	Transient upregulation by nanoparticles	–
Chemokines in BALF	Upregulation by nanoparticles	Quantity of expression: IT study ≥ IH study Persistency of expression: IT study ≥ IH study
HO-1 in BALF		
Clearance of nanoparticles	Excessive load induced delay of clearance	Biological halftime: IT study ≥ IH study

IT study: Intratracheal instillation study, IH study: Inhalation study.

Table 1 shows references in which both inhalation and intratracheal instillation studies were performed using the same nanoparticles.

Mechanism of lung disorders induced by inhaled chemicals containing nanoparticles.

In lung disorders caused by dust, phagocytosis of dust induces the infiltration of neutrophils and alveolar macrophages, and persistent or progressive inflammation is likely to cause lung injury and lead to irreversible changes, such as fibrosis and tumors. In this mechanism, alveolus macrophages englobe dust and produce chemokines and inflammatory cytokines, and inflammatory cells such as neutrophils and macrophages accumulate in the lungs through these chemokines [10]. Persistent inflammation advances the pulmonary injury with free radicals, and finally leads to fibrosis, which is based on abnormal wound repair and respiratory tumor, which is based on the genetic and epigenetic abnormality of epithelial cells [11]. Therefore the persistence of pulmonary inflammation is related to the onset and progress of lung disorders caused by respirable chemical substances. We reviewed the inflammation-related factors as predictive markers of pulmonary toxicity induced by nanoparticles.

## 2. Physicochemical Characteristics of Nanoparticles

It is important to measure the physicochemical characteristics of nanoparticles. Even if the same raw materials of nanoparticles are used, the pulmonary reactions, such as inflammation, vary greatly between laboratories according to their physicochemical differences. For example, it has recently been reported that the length of a nanoparticle is related to its toxicity. Mice were intraperitoneally exposed to two kinds of carbon nanotube (CNT) of different lengths (<5 and >20 μm) [2], and only the longer CNT induced granulomatous inflammation in the animals. Oberdorster *et al.* [3] reported that specific surface area was associated with infiltration of neutrophils in the lung. In their study, rats were intratracheally instilled with titanium dioxide (TiO_2_) particles with primary diameters of submicron and nano sizes, and the infiltration of neutrophils into the lung was higher for the nanoparticles than for the submicron particles at the same mass doses. The following physicochemical characteristics of raw materials are necessary to estimate the pulmonary toxicity of nanoparticles: (1) primary diameter; (2) agglomerated diameter in suspension; (3) specific surface area (value of Brunauer, Emmett, Teller (BET) (m^2^/g); (4) number (/g); (5) percentage of nanoparticles of more than 10 and 15 μm in length; (6) density; (7) crystalline structure; (8) components; (9) dosage (mg/rat or mg/kg); and (10) covered substance. The last three of these properties are considered in applications. Furthermore, it is important to examine the aerodynamic diameter, bulk density, geometric mean diameter and length, and average mass concentration in the exposure chamber in an inhalation study in order to calculate the initial lung burden of inhaled nanoparticles. The information about these physicochemical characteristics is essential in judging the pulmonary toxicity of nanoparticles.

## 3. Dosage

If the exposure level in an inhalation study is over a certain level, the pulmonary responses are influenced by not only the toxicity of the nanoparticle but also by the overload of the nanoparticle [12,13]. The overload of materials is due to a dysfunction of the alveolar macrophages, and this phenomenon is accompanied by a delay of the clearance of materials from the lung and the pulmonary response. It is difficult to distinguish whether the responses induced by surplus doses of nanoparticles are due to the original toxicity of nanoparticles or to the overload. Similar effects were also considered in intratracheal instillation studies, but there are big issues in such studies. It is difficult to speculate on the overload volume in intratracheal instillation studies because the additional pulmonary response is observed by the bolus shot of nanoparticles, unlike in inhalation studies [5,14], and in the pulmonary response we can not distinguish between the original toxicity, the bolus effect and overload. Considering the volume overload in inhalation studies, we think that the dose in intratracheal instillation studies should at least not be beyond the volume of delay of clearance. We [15] conducted an intratracheal instillation of 0.1, 0.2, 1 and 3 mg of TiO_2_ nanoparticles (P90) with low toxicity in rats, in which pulmonary inflammation and the delay of clearance of the TiO_2_ nanoparticles were observed at doses of 1 mg and 3 mg/rat compared with doses of 0.1 and 0.2 mg. When a dose of more than 1mg/rat of fullerene, another nanoparticle with low toxicity, was instilled to rats, persistent inflammation in rat lung was observed [16]. Therefore, if the pulmonary toxicity of nanoparticles is estimated under the same weight base, pulmonary responses at doses of 1 mg/rat (5 mg/kg) as a maximum dose may be useful, at least partially.

**Figure 1:**
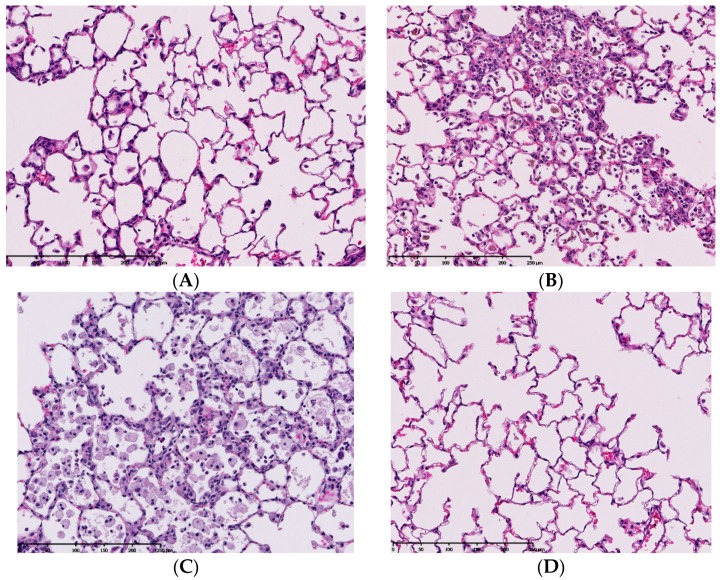
Pathological features of lung tissue in rats after intratracheal instillation of nanoparticles. Magnification 100×. (**A**) 1mg-crystalline silica-exposed lung at one week; (**B**) 1mg-TiO_2_ nanoparticle-exposed lung at one week; (**C**) 1mg-crystalline silica-exposed lung at six months; (**D**) 1mg-TiO_2_ nanoparticle-exposed lung at six months. Pulmonary inflammation in TiO_2_-exposed lung was more severe than that in crystalline silica-exposed lung at one week following intratracheal instillation. However, pulmonary inflammation in TiO_2_-exposed lung disappeared at six months and the inflammation in crystalline-exposed lung severely increased.

## 4. Observation Time

Some inhalation studies have an observation period of three months in order to observe the recovery from pathological changes [11,12]. The persistence of pathological changes should be similarly observed in intratracheal instillation studies. We performed intratracheal instillations of different mineral fibers to rats and examined lung inflammation from three days to six months [11]. Harmful respirable particles like crystalline silica and crocidolite asbestos, which are kinds of asbestos, caused persistent inflammation from the initial instillation until six months later (Figure 1). Intratracheal exposure of nickel oxide (NiO) nanoparticles induced pulmonary inflammation in rats, and the peak of inflammation was at three months post exposure [10]. Creutzenberg *et al.* [17] performed an intratracheal instillation study of TiO_2_ and a positive control made of quartz, and the number of neutrophils in the positive control increased in a time dependent fashion (until 90 days post exposure). On the other hand, when less harmful micron-sized TiO_2_ was inhaled, only a transient inflammation was observed early in the instillation. Even nanoparticles with low toxicity induce pulmonary inflammation when the observation time is less than one month. Warheit *et al.* [18] examined the pulmonary inflammation following intratracheal instillation of a variety of TiO_2_ from 24 h to three months, and there was a TiO_2_ in which pulmonary inflammation lasted at maximum for one month. Kobayashi *et al.* [19] showed that different evaluations of pulmonary toxicity by intratracheal instillation of TiO_2_ nanoparticles can be derived on the basis of observations up to one week post-instillation and those after one month post-instillation. Figure 1 shows pathological features in the lung exposed to respirable chemicals in not only the acute but also the chronic phase. Chemicals with low toxicity induced transient inflammation in the lung in the acute phase (one week), and chemicals with high toxicity induced persistent inflammation in the chronic phase (six months). Considering the persistence of pulmonary inflammation induced by nanoparticles, it is important to evaluate their pulmonary toxicity with a sufficient recovery time of three to six months.

## 5. Distribution of Nanoparticle Deposition in the Lung

**Figure 2:**
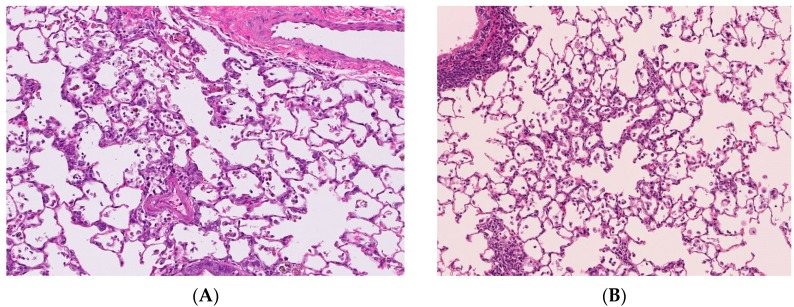
Pathological features of lung tissue. Magnification 100×. (**A**) Lung tissue following intratracheal instillation of NiO nanoparticles; (**B**) Lung tissue following inhalation of NiO nanoparticles. Distribution of infiltration of inflammatory cells in the lung were mainly centrilobular lesions in both studies.

The distribution of the deposition of nanoparticles in the lung following intratracheal instillation is the lesion in which the suspension of nanoparticles is spread, namely the centriloblar spaces, which are the neighboring alveolus lesion around the peripheral respiratory tract (Figure 2A). The infiltration of inflammatory cells such as neutrophils and macrophages with the accumulation of nanoparticles was observed in this centrilobular space after intratracheal instillation [6,20].

On the other hand, there are reports in which the distribution of infiltration of inflammatory cells in the lung was mainly in centrilobular lesions just after the end of the inhalation exposure of nanoparticles [9,21,22,23] (Figure 2B). Although the main lesions in the lung following the inhalation of nanoparticles are centrilobular lesions, as in intratracheal instillations, the inflammatory lesions were observed not only deeply in the alveolar wall but also in the subpleural area [9,22].

**Figure 3:**
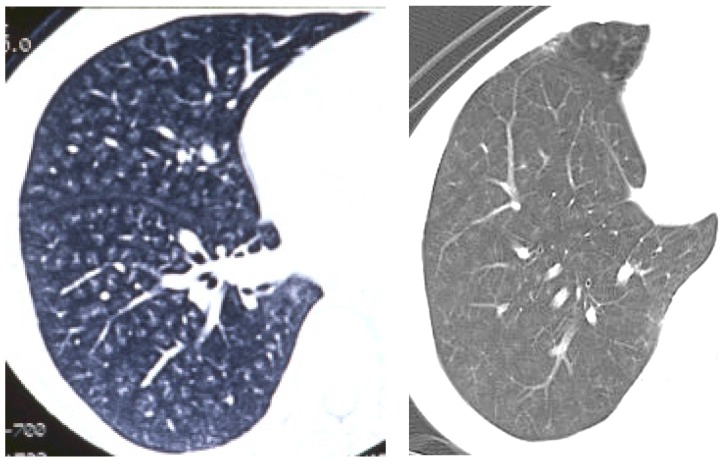
Pictures of chest in patients with welder’s lung using chest computed tomography. Nodular opacities (diffuse white opacities in the lung) were mainly observed in centrilobular lesions in the lung.

As for human data, there are some reports that exposure to welding fumes including nanoparticles induced pneumoconiosis [24,25,26], although there is no report on exposure to nanoparticles only. The distribution of welding fume deposition in the lung was in a centrilobular pattern [27] (Figure 3). Therefore the distribution of nanoparticle deposition in intratracheal instillation studies may not be very different from that in inhalation studies and human exposure. However, unless the process of dispersion of nanoparticles is performed, agglomerates of nanoparticles might be deposited in the large bronchiole and not come into the lung. It is necessary that the dispersion of nanoparticles be confirmed.

## 6. Predictive Markers of Pulmonary Toxicity

Table 2 shows the similarities and differences of inflammation as the endpoint of pulmonary toxicity between intratracheal instillation and inhalation studies. The similarities in both studies are that both inhalation and intratracheal instillation exposures induce pulmonary inflammation and related factors such as cell analysis, chemokine, proinflammatory cytokine and oxidative stress in BALF. The difference is that there is a tendency that the durability and quantity of the inflammatory response following the intratracheal instillation of nanoparticles are greater than or equal to those following the inhalation of nanoparticles, although not all papers show the same results. Detailed contents are shown in the following section.

## 7. Cell Analysis of Bronchoalveolar Lavage Fluid (BALF)

Cell analysis of BALF has been used in most papers to examine the toxicity of inhaled particles, with neutrophil cell counts and percentage of neutrophils in the BALF often being used as markers that reflect the pulmonary toxicity in both intratracheal instillation and inhalation studies (Table 2). Particles with high toxicity induced persistent neutrophil cell counts and percentage of neutrophils in the BALF following intratracheal instillation. A persistent influx of neutrophils in the lung was observed following intratracheal instillation of silica and asbestos [17,28]. Jeong *et al.* [29] found that intratracheal instillation of NiO nanoparticles induced a persistent influx of neutrophils in an animal model. We performed an intratracheal instillation study of three nanoparticles with different toxicity [6,7], NiO, TiO_2_ and cerium dioxide (CeO_2_) nanoparticles, in which NiO and CeO_2_, nanoparticles with high toxicity, induced persistent total cell and neutrophil counts and percentage of neutrophils in the BALF, and TiO_2_, a nanoparticle with low toxicity, induced only transient ones. A similar tendency was observed in our experiments with fullerene and other NiO nanoparticles. In an intratracheal instillation study, NiO at a dose of 0.2 mg/rat caused high and persistent total cell and neutrophil counts in BALF, while fullerene, a nanoparticle with low toxicity, did so transiently [16]. These neutrophil influxes in BALF corresponded to pulmonary infiltration as pathological features.

Because there are inflammatory cell types in the phenotype of macrophages, many reports showed that exposure to chemicals increased the number of macrophages in BALF, although some particles decreased the number of macrophages in a particle-induced animal model. Tan *et al.* [30] reported that intratracheal instillation of asbestos reduced the number of macrophages in BALF, and that the population of macrophages in total cells became less than 10%. Haegens *et al.* [31] reported that inhalation of asbestos decreased the number of macrophages in mice and did not change the total cell counts in BALF due to the increased number of neutrophils. Crystalline silica also decreased the number of alveolar macrophages through apoptosis [32,33]. The decrease in macrophages may have been cell death due to the toxicity of the particles. As the influx of neutrophils in the lung was observed regardless of the number of macrophages, neutrophil cell counts and percentage of neutrophils in BALF may be pulmonary toxicity predictive markers which reflect the lung inflammation.

As for quantitative pulmonary inflammation in both intratracheal instillation and inhalation studies, the neutrophil inflammation in the lung tissue in intratracheal instillation studies is higher than that in inhalation studies. According to Baisch *et al.* [5], there were significantly more neutrophils in BALF following the intratracheal instillation of TiO_2_ nanoparticles compared to inhalation, even though the initial lung burdens were the same. Silva *et al.* [8] also showed that the infiltration of inflammatory cells following the intratracheal instillation of multiwall carbon nanotube (MWCNT) was greater than that following inhalation of MWCNT. Our experiments with NiO and TiO_2_ nanoparticles showed a similar tendency [6].

As for persistency of inflammation, neutrophil inflammation in the lung tissue in intratracheal instillation studies is equal to or more persistent than that in inhalation studies. A more persistent inflammation in BALF following the intratracheal instillation of NiO nanoparticles in the lung was observed in comparison to inhalation, even though the initial lung burdens were the same. The bolus effect may have resulted in the values of the data from intratracheal instillation being higher and more persistent than those from inhalation, but the bolus effect can not be avoided in an intratracheal instillation. Although the bolus effects are largely observed in the acute phase after exposure, the toxicity of nanoparticles may be estimated in the chronic phase, such as three months or six months postexposure, when the bolus effect is minimalized.

## 8. Proinflammatory Cytokines and Chemokines

Proinflammatory cytokines and the cytokine-induced neutrophil chemoattractant (CINC) families, including macrophage inflammatory protein-2 (MIP-2), chemokine for neutrophil and macrophages, are reported to be related to pulmonary inflammation induced by inhaled chemicals in both approaches.

The proinflammatory cytokines interleukin-1 (IL-1), IL-6 and tumor necrosis factor (TNF) were reported to be upregulated in the acute phase in animal models exposed to inhaled materials. Exposure to crystalline silica and TiO_2_ nanoparticles induced the concentration of proinflammatory cytokines in BALF in the acute phase only [34,35,36], and exposure to micron-sized and nano-sized crystalline silica also induced transient IL-1 and TNF concentration [34], and exposure to NiO nanoparticles transiently induced IL-1, IL-6, and TNF concentration in lung tissue in the acute phase [37]. These transient increases in the concentration of proinflammaory cytokines may be the trigger to sustained pulmonary inflammation, although the setting of the observation period for a transient expression may make it difficult to use proinflammatory cytokines as a biomarker of inflammation.

**Figure 4:**
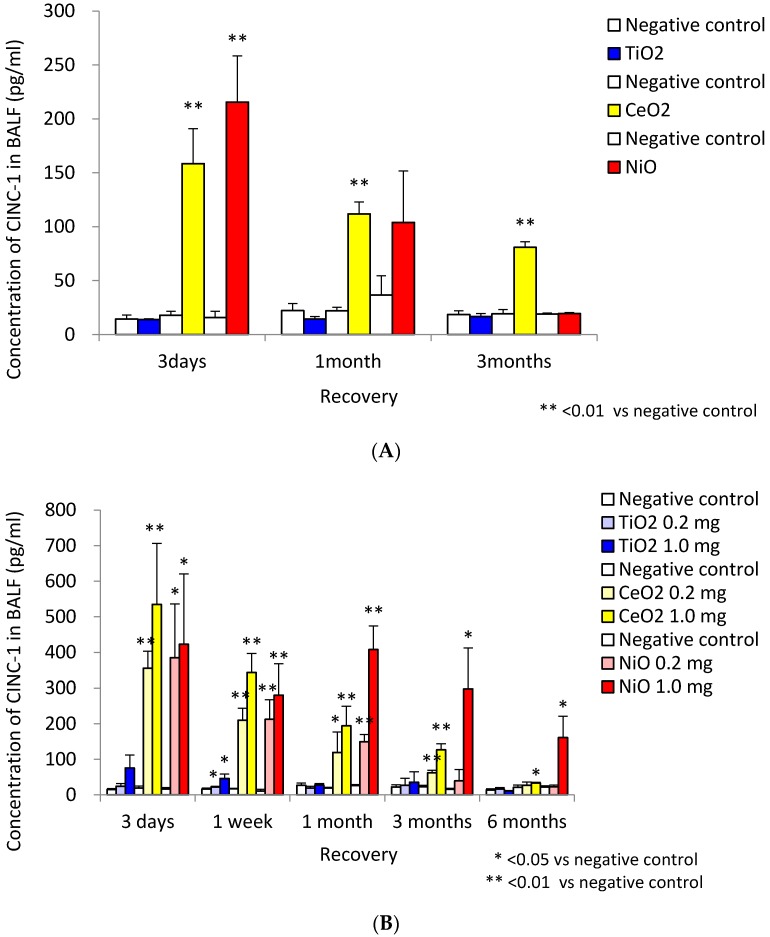
Concentration of CINC-1 in BALF. (**A**) data following inhalation of NiO, CeO_2_ and TiO_2_ nanoparticles; (**B**) data following intratracheal instillation of NiO, CeO_2_ and TiO_2_ nanoparticles. Inhalation exposure of NiO and CeO_2_ nanoparticles, but not TiO_2_ nanoparticles, induced the expression of CINC-1. Intratracheal instillation of NiO, CeO_2_ induced persistent expression of CINC-1, whereas TiO_2_ induced transient expression. (This research was originally published in [6] and [7]).

In a study of chemokines, Baisch *et al.* [5] examined the gene expression of monocyte chemoattractant protein-1 (MCP-1) and CINC-3 (also known as MIP-2) in the lung following inhalation and intratracheal instillation of TiO_2_. Both exposures induced transient increases in the level of MCP-1 and CINC-3 in the lung tissue accompanied by transient neutrophil infiltration. We also performed inhalation and intratracheal instillation studies of NiO, CeO_2_ and TiO_2_ [6,7]. Inhalation exposure of NiO and CeO_2_ nanoparticles, which induced neutrophil inflammation, induced the expression of CINC-1 and CINC-2 in BALF (Figure 4A). Inhalation exposure of TiO_2_ nanoparticles did not induce such effects. Intratracheal instillation of NiO and CeO_2_, which induced persistent neutrophil inflammation, induced the persistent expression of CINC-1 and CINC-2, whereas TiO_2_, which induced transient inflammation, induced transient expression in BALF (Figure 4B). Gustafsson *et al.* [36] reported that intratracheal exposure of TiO_2_ nanoparticles (P25) increased the concentration of CINC-1 in BALF transiently, and persistent neutrophil inflammation was observed through 90 days post exposure, during which time its peak was at 16 days and it came close to control level at 90 days. Adamcakova-Dodd *et al.* [38] reported that the concentration of MIP-1α and MCP-1 in BALF was not increased in mice exposed to sub-acute and sub-chronic inhalation of zinc oxide (ZnO) nanoparticles with low toxicity.

Keratinocyte derived chemokines (KC) have been reported to increase persistently in lung following intratracheal instillation of quartz. Seiffert *et al.* [39] reported that the intratracheal instillation of silver nanoparticles induced a transient KC concentration in BALF, and that the chemokine expression pattern was accompanied by neutrophil influx in the lung. The inhalation of ZnO nanoparticles with low toxicity did not induce neutrophil infiltration or an increased KC level in BALF [38].

Chemokines such as the CINC family, MIP, MCP and KC reflect the neutrophil influx in the lung, suggesting that these chemokines may be predictive markers of pulmonary toxicity.

## 9. Oxidative Stress Markers

Many studies have reported that oxidative stress, such as free radicals, antioxidants, and oxidative damage, was induced by nanoparticles. Among these oxidative stress markers, heme oxygenase-1 (HO-1), an antioxidant, may become a predictive marker of pulmonary toxicity. There are reports on the upregulation of HO-1 in the lung exposed to nanoparticles following not only inhalation but also intratracheal instillation [6,7,40]. A short term inhalation exposure of diesel engine exhaust, which is known as particulate air pollution with adverse health effects, induced the expression of HO-1 mRNA in the lung at 18 h post recovery. Sun *et al.* [40] reported that intratracheal exposure to TiO_2_ nanoparticles induced not only persistent pulmonary inflammation but also persistent gene expression of HO-1 in the lung. In our inhalation and intratracheal instillation studies of three nanoparticles, of which NiO and CeO_2_ had high toxicity and TiO_2_ had low toxicity, the inhalation of NiO and CeO_2_ increased the concentration of HO-1 in BALF, but TiO_2_ did not, while the intratracheal instillation of NiO and CeO_2_ caused a persistent increase in the concentration of HO-1 in BALF and TiO_2_ caused a transient increase [6,7].

Although there are many reports on glutathione level in the lung induced by nanoparticles, some studies have reported an upregulation of glutathione levels induced by nanoparticles, while others have reported a downregulation. Srinivas *et al.* [41] reported that inhalation exposure of CeO_2_ nanoparticles reduced the concentration of glutathione (GSH) in the lung during a 14-day recovery time. The inhalation of singlewall carbon nanotube (SWCNT) also reduced the GSH level in the lung of mice [9]. On the other hand, there are some studies that have shown an increase in glutathione from exposure of nanoparticles. Braakhuis *et al.* [42] reported that inhalation of silver nanoparticles for four consecutive days induced a transient increase in glutathione concentration in the lung, which corresponded to transient neutrophil infiltration. Acute and chronic inhalation of cadmium oxide (CdO) nanoparticles increased the concentration of glutathione in the lung [42]. As for intratracheal instillation studies, Zhu *et al.* reported [43] that the content of GSH decreased in nano-sized and submicron-sized Fe_2_O_3_ instilled rats at one to seven post-instilled days and then recovered to control level at day 30. The different responses of glutathione induced by nanoparticles may make it difficult to use as a predictor of pulmonary toxicity.

Some papers have shown that oxidative stress induced by nanoparticles increased the level of malondialdehyde (MDA). Srinivas *et al.* [41] reported that inhalation exposure of CeO_2_ nanoparticles induced neutrophil infiltration in the lung during the observation time (14 days post exposure) and increased the concentration of MDA in the lung persistently. Inhalation of SWCNT also induced MDA level in the lung of mice [9]. Zhu *et al.* [43] reported that a neutrophil influx was observed in nano-sized and submicron-sized Fe_2_O_3_ instilled rats at one to seven post-instilled days, and it then recovered to control level at day 30, although the MDA levels in the lung exposed to Fe_2_O_3_, except for a low dose of nanoparticles, increased up to 30 days. Because MDA level tends to last even if inflammation resolves, it may be difficult to use MDA alone to predict pulmonary toxicity.

### Biological Halftimes of Nanoparticles

There are some reports that the biological halftimes of materials following intratracheal instillation are the same as or longer than those following inhalation [6,20]. Baisch *et al.* [5] compared the clearance of TiO_2_ nanoparticles in the lung following inhalation and intratracheal instillation under the same initial lung burden. Two thirds of the TiO_2_ nanoparticles were cleared at seven days after the end of inhalation, but in the intratracheal instillation study most of the TiO_2_ did not clear from the lung at seven days. We performed four-week inhalation and intratracheal instillation studies of potassium hexatitanate, which is not a nanoparticle, and examined its biopersistence [44]. Its biological halftimes following inhalation and intratracheal instillation were 2.3 and 3.1 months, respectively. Silver *et al.* [8] examined the lung burden induced by MWCNT at one day and 21 days following intratracheal instillation and inhalation, and found no clearance of MWCNT in either approach, although the halftimes were not shown. The bolus effect may have caused the result that there was a longer delay of clearance of nanoparticles in the intratracheal instillation than in the inhalation. The avoidance of an excessive dosage in an intratracheal instillation of nanoparticles will diminish the delayed clearance of nanoparticles in the lung. If the dosage in the intratracheal instillation of nanoparticles is regulated, pulmonary responses caused by the difference in clearance of nanoparticles can be kept to a minimum level. Oyabu *et al.* [15] showed that if the dose of TiO_2_ nanoparticles for an intratracheal instillation is less than 1 mg/rat, the delayed clearance of nanoparticles can be minimized. The regulation of dosage may vary according to the density of the material, but it is important to consider that a delay of the clearance from an excessive dosage may cause additional responses by nanoparticles.

## 10. Conclusions

We reviewed the effectiveness of intratracheal instillation studies for the estimation of the hazard of nanoparticles. A difference in pulmonary inflammation was observed between high and low toxicity nanoparticles in the intratracheal instillation studies, as in the inhalation studies. Among the endpoints of pulmonary toxicity, the kinetics of neutrophil counts, percentage of neutrophils, and chemokines for neutrophils and macrophages, HO-1 in BALF, reflected pulmonary inflammation, suggesting that these may be useful as predictive markers of pulmonary toxicity in both types of studies. If a comparison is made of pulmonary inflammation between intratracheal instillation and inhalation studies under the same initial lung burden, there is a tendency that the inflammatory response following the intratracheal instillation of nanoparticles is greater than or equal to that following the inhalation of nanoparticles. Taken together, we suggest that intratracheal instillation studies can be useful for ranking the hazard of nanoparticles based on pulmonary inflammation.
